# Improving pediatric hip fracture detection using deep learning: multicenter validation and clinical reader study

**DOI:** 10.1097/JS9.0000000000005138

**Published:** 2026-03-18

**Authors:** Tongtong Huo, Xiaoliang Chen, Pengran Liu, Jin Liu, Zineng Yan, Jiaming Yang, Songxiang Liu, Lin Lu, Jiayao Zhang, Jia Shao, Wei Wu, Mingdi Xue, Zhewei Ye

**Affiliations:** a School of Electronic Information, Wuhan University of Science and Technology, Wuhan, P.R. China; b Department of Orthopedics, Union Hospital, Tongji Medical College, Huazhong University of Science and Technology, Wuhan, P.R. China; c Department of Orthopedics, People’s Hospital of Ningxia Hui Autonomous Region, Ningxia Medical University, Yinchuan, P.R. China; d Department of Orthopedics, Renmin Hospital of Wuhan University, Wuhan, P.R. China; e Department of Orthopedics, Fuzhou University Affiliated Provincial Hospital, Fuzhou, P.R. China; f Department of Orthopedics, Department of Spine Surgery, Henan Provincial People’s Hospital, Zhengzhou, P.R. China

**Keywords:** artificial intelligence, deep learning, diagnostic accuracy, hip radiograph, pediatric femoral neck fracture

## Abstract

**Background:**

To develop and evaluate a deep learning model for automated localization and diagnosis of femoral neck fractures in children under 8 years of age using hip radiographs.

**Materials and Methods:**

This retrospective multicenter study included 794 hip radiographs from 640 pediatric patients (median age, 4.1 years; 62.5% male) collected from four tertiary hospitals between June 2013 and December 2024. A YOLOv11-based object detection model was trained on 712 radiographs and externally validated on 82 radiographs. Diagnostic performance was measured by area under the receiver operating characteristic curve (AUROC), sensitivity, and specificity. A multi-reader study was conducted using the external test set, where five physicians (two senior radiologists, one junior radiologist, two emergency physicians) interpreted radiographs with and without AI assistance. Statistical analysis included DeLong’s test, McNemar tests, and Fleiss’ κ.

**Results:**

The model achieved AUROCs of 0.911 (95% CI: 0.864–0.949) on the internal test set and 0.873 (95% CI: 0.792–0.935) on the external test set. Sensitivity and specificity were 84.9% and 85.5% internally, and 80.8% and 91.1% externally. Among junior readers, AI assistance significantly improved diagnostic accuracy (mean ΔAUROC = + 0.083; *P* = 0.007) and interobserver agreement (κ from 0.49 to 0.61). The model localized fractures in real time with a mean inference time of 56.2 ms.

**Conclusion:**

A YOLOv11-based deep learning model accurately detected femoral neck fractures in children and significantly improved diagnostic accuracy and consistency among less experienced readers. These findings support its integration as a real-time assistive tool in pediatric trauma care.

## Introduction

Pediatric femoral neck fractures are rare but clinically significant injuries, accounting for less than 1% of all pediatric fractures[1]. Despite their low incidence, these fractures are associated with serious complications such as avascular necrosis, premature physeal closure, coxa vara, and limb length discrepancy^[^2,3^]^. Early and accurate diagnosis is essential to prevent these sequelae, particularly within the narrow therapeutic window of early childhood.

Accurate detection in this age group remains challenging due to both anatomical immaturity and imaging limitations. The proximal femur in young children is incompletely ossified, and normal developmental structures – such as cartilaginous femoral heads, open physes, and apophyseal centers – can mimic fracture lines on radiographs^[^4,5^]^. These features may closely resemble subtle or nondisplaced fractures, leading to misinterpretation even among experienced pediatric radiologists and orthopedic surgeons^[^6,7^]^. In general hospitals and emergency departments, where pediatric musculoskeletal expertise may be limited, the risk of diagnostic error is further heightened^[^8,9^]^. A landmark study reported that up to 80% of diagnostic errors in emergency settings involved missed fractures, with radiographic misinterpretation accounting for the majority.[10] Although CT and MRI provide supplementary information, plain radiography remains the first-line imaging modality due to its accessibility, speed, and low radiation exposure.

Recent advances in artificial intelligence (AI), particularly deep learning–based object detection, have shown promising results in musculoskeletal imaging, especially for detecting fractures in adults and common pediatric sites such as the wrist and elbow^[^11,12^]^. However, these models have primarily been developed for high-prevalence and anatomically straightforward injuries^[^13–16^]^. In contrast, rare yet high-stakes injuries like pediatric femoral neck fractures pose unique challenges due to their morphological complexity and lower incidence[17]. Furthermore, few studies have assessed AI model performance in clinically realistic scenarios involving human–AI collaboration or workflow integration^[^18–20^]^.

To address this unmet need, we developed and validated a YOLOv11-based object detection model to localize femoral neck fractures on pediatric hip radiographs. YOLOv11 was selected for its real-time inference capability, strong localization performance, and robustness in detecting small or complex anatomical targets^[^21–23^]^. We further conducted a multi-reader study to assess the effect of AI assistance on diagnostic performance and interobserver agreement among clinicians with varying experience levels. A qualitative error analysis was also performed to characterize failure modes and inform future improvements.

## Materials and methods

### Study design and ethical approval

This retrospective multicenter diagnostic accuracy study was conducted across four tertiary care hospitals in China from June 2013 to December 2024. Institutional review board approval was obtained at all participating centers, with a waiver of informed consent granted due to the retrospective nature of the study and the exclusive use of anonymized radiographic data. The study adhered to the Declaration of Helsinki and complied with national guidelines regarding the secondary use of clinical imaging data. This work has been reported in line with the STROCSS criteria[24].

### Data curation

Eligible participants were children under 8 years of age who underwent hip radiography for suspected traumatic injury between June 2013 and December 2024. Imaging was clinically indicated based on focal symptoms such as limping, refusal to bear weight, localized tenderness, or reported fall, rather than for routine screening.

Radiographs were retrospectively retrieved from the institutional radiology information systems using keyword-based search filters (e.g., “hip,” “fracture,” “trauma,” “child”). Inclusion criteria were: (1) radiographs acquired during the initial trauma evaluation; (2) digital images of sufficient diagnostic quality; (3) fracture status confirmed by CT or short-term clinical/radiographic follow-up; (4) one radiograph per trauma episode per patient. Exclusion criteria included: (1) poor image quality (e.g., motion blur, cropped field of view, underexposure); (2) non-traumatic or follow-up indications (e.g., developmental dysplasia, postoperative assessment); (3) incomplete clinical or imaging data.

The development set comprised 712 radiographs from 570 unique patients across three institutions, and was partitioned into training (*n* = 499), tuning (*n* = 71), and internal test (*n* = 142) subsets using a stratified 7:1:2 ratio based on fracture status. An external test set was independently collected from a fourth hospital, comprising 82 radiographs from 70 patients using identical eligibility criteria. Among them, 12 patients contributed both anteroposterior and frog-leg lateral views. Evaluation was performed on a per-radiograph basis; therefore, both views were included as separate inputs to assess the model’s performance across different projections.

HIGHLIGHTS

- First multicenter AI validation for pediatric femoral neck fractures.
- Largest dataset curated: 794 radiographs from 640 patients.
- Yolov11 model achieved an external AUROC of 0.873 (56 ms inference).
- AI and human readers demonstrate distinct, complementary error patterns.
- AI significantly improved junior clinicians’ accuracy (δAUROC + 0.083).

Image quality was independently verified by two pediatric radiologists (10 and 12 years of experience). Radiographs with motion artifacts, incomplete visualization of the proximal femur, or insufficient contrast were excluded. All accepted images were normalized for intensity and resized to 640 × 640 pixels prior to model input.

### Reference standard

Fracture status (positive or negative) was determined by two board-certified pediatric orthopedic surgeons (18 and 6 years of clinical experience). The reference standard was established prior to model training and was completely independent of the AI model’s outputs and the subsequent reader study results. Adjudicators were blinded to all AI predictions and reader interpretations. For each patient, diagnostic adjudication was based on available clinical data, including initial radiographs, CT scans (if obtained), and follow-up imaging within 2–4 weeks. Fracture-positive cases were defined by definitive radiographic evidence on the initial study or confirmatory findings on CT. Fracture-negative cases required both normal initial imaging and absence of delayed diagnosis on follow-up.

Although final adjudication was conducted by orthopedic surgeons, all decisions incorporated detailed image review and clinical correlation, consistent with standard pediatric trauma workflows where orthopedic teams typically lead diagnosis and management. All included cases were quality-screened by pediatric radiologists to ensure technical adequacy.

Annotations were generated prior to and independently of the AI model. Bounding boxes were created for fracture-positive images using the VGG Image Annotator (VIA, version 2.0.10) by trained imaging analysts and reviewed by both orthopedic surgeons. Final annotations reflected consensus on both fracture presence and precise localization. In cases of initial disagreement or diagnostic uncertainty, a senior pediatric radiologist (15 years of experience) was consulted to provide multidisciplinary input and reach a final consensus. No cases with unresolved disagreement were included. Fracture-negative radiographs were assigned no bounding boxes.

To minimize inter-reader variability, all annotations were finalized prior to model training and reviewed for consistency across institutions and age groups.

### Model development

A deep learning object detection model based on YOLOv11 was developed to detect pediatric femoral neck fractures on hip radiographs. YOLOv11 was selected for its state-of-the-art performance in small-object localization and real-time inference, making it well suited for detecting subtle fractures in immature skeletal anatomy. Compared to prior versions such as YOLOv5 and YOLOv8, YOLOv11 incorporates architectural enhancements – including Cross-Stage Partial (C2f) modules, an improved Spatial Pyramid Pooling Fast (SPPF) block, and a decoupled detection head – that enhance multi-scale feature representation and optimize classification-localization decoupling.

All radiographs were resized to 640 × 640 pixels and preprocessed with intensity normalization and standard data augmentation. Model output consisted of bounding boxes with fracture class labels and confidence scores ranging from 0 to 1. A prediction was considered correct if it met the intersection-over-union (IoU ≥ 0.5) and confidence (≥ 0.25) thresholds determined via tuning set optimization.

Full implementation details, including network architecture, training hyperparameters, and ablation strategies, are provided in Supplemental Digital Content Section S1, available at: http://links.lww.com/JS9/H118, and Supplemental Digital Content Tables 1–2, available at: http://links.lww.com/JS9/H118.

### Observer study

To assess the clinical utility of the AI model, we conducted a fully crossed, two-session multi-reader observer study using the external test set. Five board-certified physicians participated, including two senior radiologists (15 and 12 years of experience), one junior radiologist (2 years), and two emergency physicians (5 and 3 years). All participants routinely interpret pediatric trauma radiographs, although none had formal subspecialty training in pediatric musculoskeletal imaging.

Each reader independently reviewed all 82 radiographs under two conditions: (1) unaided session: Radiographs were interpreted without AI assistance. (2) AI-assisted session: Model-generated bounding boxes and confidence scores were overlaid on the images. The session order was fixed (unaided followed by AI-assisted) to avoid bias introduced by AI exposure. A 4-week washout period was implemented to minimize recall bias. The presentation order of radiographs was independently randomized for each session.

Readers were blinded to fracture prevalence, reference standards, and model outputs during the unaided session. For each case, they provided a binary diagnosis (fracture-positive or negative) and rated their diagnostic confidence using a 5-point Likert scale (1 = very uncertain, 5 = very confident).

### Statistical analysis

Model and reader performance were evaluated on a per-radiograph basis. For both the AI model and each human reader, the following diagnostic metrics were calculated: area under the receiver operating characteristic curve (AUROC), sensitivity, specificity, positive predictive value (PPV), negative predictive value (NPV), and accuracy. A prediction was considered a true positive if the bounding box overlapped the reference annotation at an intersection-over-union ≥ 0.5 and had a confidence score ≥ 0.25, consistent with thresholds determined from the tuning set.

To evaluate the impact of AI assistance, the differences in AUROC between unaided and AI-assisted sessions were assessed using DeLong’s test[25] for correlated ROC curves for each individual reader. For group-level analysis, the mean AUROC values were calculated, and the improvement was analyzed using a paired approach. McNemar’s test was used to assess changes in sensitivity and specificity. Changes in reader confidence (Likert scores) were analyzed using the Wilcoxon signed-rank test.

Interobserver agreement among the five readers was assessed using Fleiss’ κ statistic, with values interpreted as follows: 0.41–0.60 (moderate agreement) and 0.61–0.80 (substantial agreement).

All analyses were performed in Python 3.9 using the scikit-learn, SciPy, and statsmodels libraries. A two-sided P value < 0.05 was considered statistically significant.

## Results

### Patient characteristics

**Figure 1.**
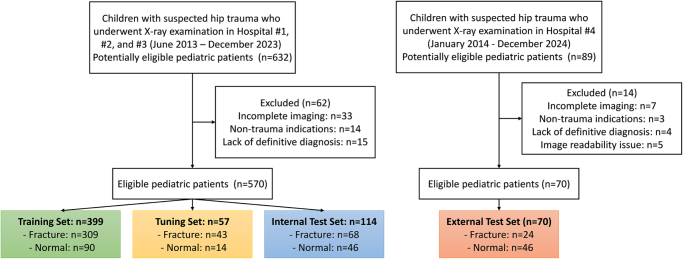
Study cohort flowchart. Flowchart illustrating patient selection, inclusion and exclusion criteria, and data partitioning across the development and external test datasets. A total of 794 radiographs from 640 pediatric patients were included: 570 patients were assigned to the development dataset (for training, tuning, and internal testing) and 70 to the external test set.

A total of 794 hip radiographs from 640 pediatric patients (median age, 4.1 years; interquartile range, 2.6–5.8 years; 62.5% male) were included from four tertiary centers. The development dataset comprised 712 radiographs from 570 patients, while the external test set consisted of 82 radiographs from 70 patients (Fig. 1).

The prevalence of femoral neck fractures was 59.6% (68/114) in the internal test set and 34.3% (24/70) in the external test set. There were no statistically significant differences in age, sex, or image laterality across the training, tuning, and test subsets (Table 1).

**Table 1:** Demographic and clinical characteristics of the study population.

Characteristic	Training set (*n* = 399)	Tuning set (*n* = 57)	Internal test Set (*n* = 114)	External test set (*n* = 70)	*P*-value
Imaging vendor	GE Definium 6000 (34%), Siemens Ysio Max (30%), Philips DigitalDiagnost C90 (26%), Other (15%)			GE Definium(40), UIH uDR 780i (40%), Other (20%)	
Median age (IQR), years	4.1 (2.5–5.6)	4.3 (2.7–5.9)	4.2 (2.6–5.8)	4.2 (2.7–5.9)	0.070
Male sex	252 (63.2)	33 (57.9)	73 (64.0)	42 (60.0)	0.870
Fracture-positive patients	309 (77.4)	43 (75.4)	68 (59.6)	24 (34.3)	<0.001
Number of radiographs	499	71	142	82	
Frog-leg lateral views	89 (22.3)	16 (28.1)	37 (32.5)	12 (17.1)	0.060
Right-side radiographs	264 (52.9)	36 (50.7)	77 (54.2)	42 (51.2)	0.936
Left-side radiographs	235 (47.1)	35 (49.3)	65 (45.8)	40 (48.8)	0.936

Patient numbers are reported in the column headers (e.g., *n* = 399), while the number of radiographs is shown separately in the row “Number of radiographs.” Multiple radiographs per patient were allowed in the development set. Categorical variables are reported as counts with percentages in parentheses. Age is presented as median and interquartile range (IQR). “Imaging vendor” refers to the specific radiography system used at each institution. *P*-values were calculated using the Kruskal–Wallis test for continuous variables and the chi-square test for categorical variables. A *P*-value < 0.05 was considered statistically significant.

### Model performance

**Figure 2.**
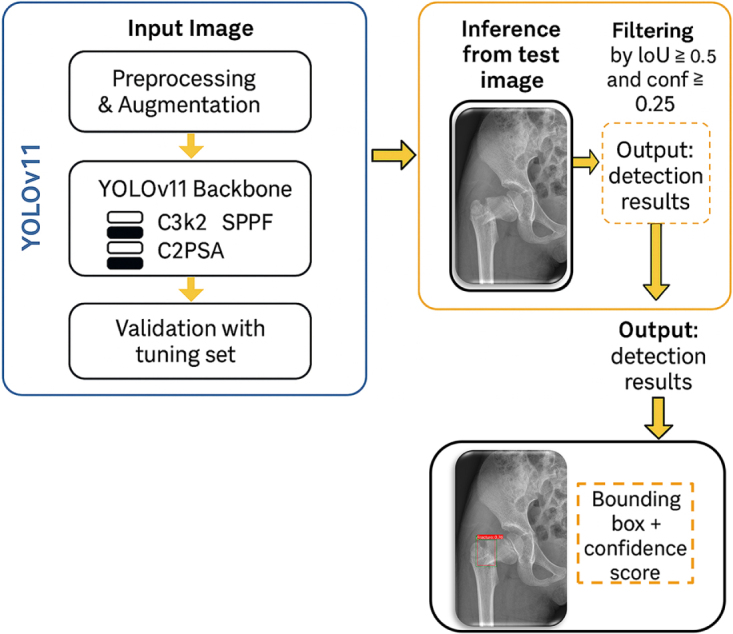
AI model architecture and workflow. Schematic representation of the YOLOv11-based object detection system for pediatric femoral neck fracture detection on hip radiographs. The training pipeline (left, blue box) includes image preprocessing, augmentation, and supervised training using a YOLOv11 backbone integrating C3k2, SPPF, and C2PSA modules, with validation performed on a tuning set. The inference process (center, orange box) evaluates test radiographs and generates bounding boxes with associated confidence scores. Final predictions are filtered using an intersection-over-union (IoU) threshold ≥ 0.5 and a confidence threshold ≥ 0.25. The output detection results are displayed in the rightmost black box. AI, artificial intelligence; IoU, intersection over union.

The YOLOv11-based object detection model demonstrated high diagnostic accuracy on both internal and external test sets (Fig. 2). On the internal test set (*n* = 142), the model achieved an AUROC of 0.911 (95% CI: 0.864–0.949), with a sensitivity of 84.9% (62/73), a specificity of 85.5% (59/69), a PPV of 86.1% (62/72), an NPV of 84.3% (59/70), and an overall accuracy of 85.2% (121/142).

**Figure 3.**
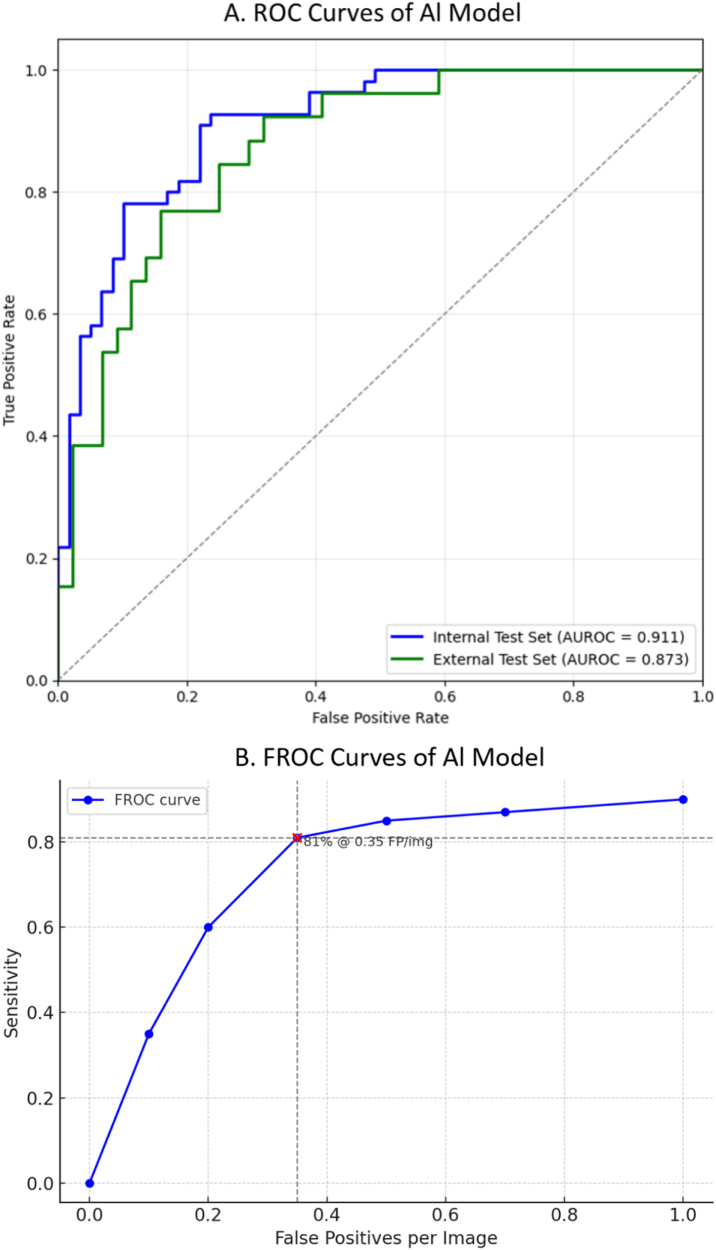
ROC and FROC curves for model performance. (A) ROC curves demonstrating AI model performance on the internal and external test sets. The model achieved AUROC values of 0.911 (internal) and 0.873 (external). (B) FROC curve for the external test set, showing sensitivity as a function of false positives per image. At 0.35 false positives per image, sensitivity reached 0.81. AI, artificial intelligence; AUROC, area under the ROC curve; FROC, free-response receiver operating characteristic; ROC, receiver operating characteristic.

**Figure 4.**
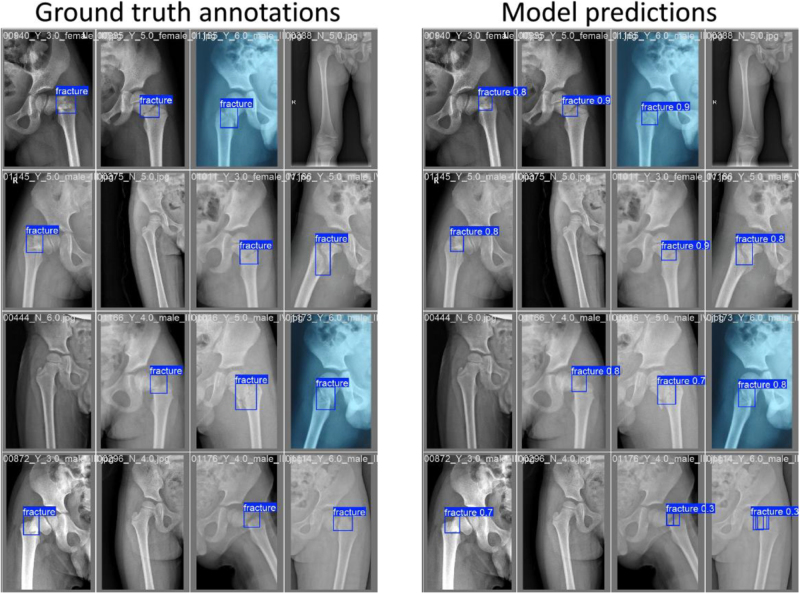
Qualitative comparison of GT and model predictions. Representative examples comparing GT annotations and AI model predictions on pediatric hip radiographs. Left column: GT labels by pediatric orthopedic surgeons. Blue boxes indicate confirmed femoral neck fractures; non-fracture radiographs are shown without annotations. Right column: AI model predictions on the same radiographs. Bounding boxes are shown with associated confidence scores for detected fractures. AI, artificial intelligence; GT, ground truth.

On the external test set (*n* = 82), the AUROC was 0.873 (95% CI: 0.792–0.935), with a sensitivity of 80.8% (21/26), a specificity of 91.1% (51/56), a PPV of 81.2% (21/26), an NPV of 86.0% (51/56), and an accuracy of 87.8% (72/82) (Table 2; Fig. 3A). Free-response receiver operating characteristic analysis showed a sensitivity of 0.81 at 0.35 false positives per image (Fig. 3B). The model’s mean inference time was 56.2 ± 4.8 ms per image. Examples of ground truth annotations and model predictions are shown in Figure 4.

**Table 2:** Performance of the YOLOv11 model on internal and external test sets.

Metric	Internal test set (*n* = 142)	External test set (*n* = 82)
Sensitivity	84.9% (62/73) [75.0–91.4]	80.8% (21/26) [62.1–91.5]
Specificity	85.5% (59/69) [75.3–91.9]	91.1% (51/56) [80.7–96.1]
PPV	86.1% (62/72) [76.3–92.3]	81.2% (21/26) [64.7–91.1]
NPV	84.3% (59/70) [74.0–91.0]	86.0% (51/56) [73.8–93.0]
Accuracy	85.2% (121/142) [78.4–90.1]	87.8% (72/82) [79.0–93.2]
AUROC	0.911 [0.864–0.949]	0.873 [0.792–0.935]
F1 score	85.4% [77.5–93.3]	80.8% [68.4–93.2]
Loc Acc	84.9% (62/73) [75.0–91.4]	80.8% (21/26) [62.1–91.5]

AUROC, area under the receiver operating characteristic curve; Loc Acc, localization accuracy; NPV, negative predictive value; PPV, positive predictive value.

Sensitivity, specificity, PPV, NPV, accuracy, and localization accuracy are reported as percentages, with corresponding numerators/denominators in parentheses and 95% confidence intervals (CIs) in brackets. AUROC and F1 scores are presented with 95% CIs. A radiograph was classified as fracture-positive if at least one predicted bounding box overlapped with the reference annotation at an intersection-over-union (IoU) ≥ 0.5 and had a model confidence score ≥ 0.25, consistent with the criteria defined in the Methods. Loc Acc was defined as the proportion of fracture-positive radiographs in which at least one predicted bounding box met both the IoU and confidence thresholds with the ground truth.

### Reader performance

Five clinicians participated in the observer study using the external test set. During the unaided session, individual AUROC values ranged from 0.711 to 0.874. The two senior radiologists (R1 and R2) demonstrated the highest baseline performance (AUROC > 0.86), whereas the junior radiologist and emergency physicians showed lower diagnostic accuracy.

**Figure 5.**
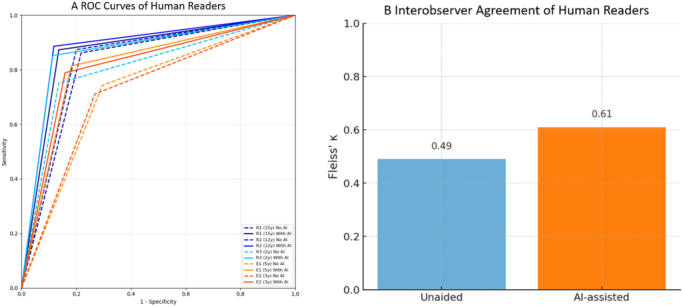
Reader performance and interobserver agreement. (A) ROC curves comparing diagnostic performance of five clinicians under unaided (dashed lines) and AI-assisted (solid lines) conditions. R1, R2, and R3 denote radiologists with 15, 12, and 2 years of experience, respectively. E1 and E2 are emergency physicians with 5 and 3 years of experience. Performance gains with AI were most pronounced among less experienced readers. (B) Interobserver agreement measured by Fleiss’ κ statistic. Agreement increased from 0.49 (moderate) in the unaided session to 0.61 (substantial) in the AI-assisted session, indicating enhanced consistency across readers. AI, artificial intelligence; ROC, receiver operating characteristic; κ, Fleiss’ kappa coefficient.

With AI assistance, all junior readers exhibited improved performance. Statistically significant AUROC gains were observed for each junior reader individually (*P* = 0.011, 0.038, and 0.045, respectively; Table 3), and the group-level mean AUROC increased from 0.735 to 0.818 (*P* = 0.007). Sensitivity improved by 7.7% to 15.4%, while specificity increased by 6.8% to 9.1% (Table 3; Fig. 5A).

**Table 3:** Reader performance with and without AI assistance.

Metric	R1	R2	R3	E1	E2	Mean (Junior Readers)
Clinical role	Senior radiologist	Senior radiologist	Junior radiologist	Emergency physician	Emergency physician	/
Experience (years)	15	12	2	5	3	3.3
AUROC (unaided)	0.862 [0.77–0.94]	0.874 [0.79–0.95]	0.751 [0.63–0.86]	0.743 [0.61–0.85]	0.711 [0.58–0.83]	0.735
AUROC (AI-assisted)	0.873 [0.78–0.95]	0.886 [0.80–0.96]	0.852 [0.75–0.93]	0.814 [0.70–0.90]	0.789 [0.68–0.88]	0.818
∆AUROC	+0.011	+0.012	+0.101	+0.071	+0.078	0.083
*P*-value	0.685	0.692	0.008	0.032	0.036	0.005
Sensitivity (unaided)	84.6% (22/26) [70.7–98.5]	88.5% (23/26) [76.2–100.0]	73.1% (19/26) [56.1–90.1]	69.2% (18/26) [51.5–86.9]	65.4% (17/26) [47.1–83.7]	69.20%
Sensitivity (AI-assisted)	84.6% (22/26) [70.7–98.5]	88.5% (23/26) [76.2–100.0]	84.6% (22/26) [70.7–98.5]	76.9% (20/26) [60.7–93.1]	80.8% (21/26) [65.7–95.9]	80.80%
∆Sensitivity	0.0	0.0	+11.5%	+7.7%	+15.4%	11.60%
*P*-value	0.999	1.000	0.034	0.089	0.031	**/**
Specificity (unaided)	86.4% (48/56) [77.4–95.4]	90.9% (51/56) [83.4–98.4]	75.0% (42/56) [63.7–86.3]	77.3% (43/56) [66.3–88.3]	72.7% (41/56) [61.0–84.4]	75.00%
Specificity (AI-assisted)	88.6% (50/56) [80.3–96.9]	90.9% (51/56) [83.4–98.4]	84.1% (47/56) [74.5–93.7]	84.1% (47/56) [74.5–93.7]	81.8% (46/56) [71.7–91.9]	83.30%
∆Specificity	+2.2%	0.0	+9.1%	+6.8%	+9.1%	8.30%
*P*-value	0.802	1.000	0.044	0.046	0.058	**/**
Accuracy (unaided)	85.4% (72/82) [76.4–92.3]	89.4% (75/82) [80.9–95.0]	74.4% (61/82) [63.8–83.3]	74.4% (61/82) [63.8–83.3]	70.7% (58/82) [59.9–80.0]	73.2%
Accuracy (AI-assisted)	86.6% (73/82) [77.8–92.9]	89.4% (75/82) [80.9–95.0]	84.1% (69/82) [74.8–91.3]	80.5% (66/82) [70.6–88.4]	81.7% (67/82) [71.9–89.7]	82.1%
ΔAccuracy	1.20%	0.00%	9.70%	6.10%	11.00%	8.90%
PPV (unaided)	73.3% [55.6–85.8]	82.1% [64.4–92.1]	57.6% [40.8–72.8]	58.1% [40.8–73.6]	53.1% [36.4–69.1]	56.2%
PPV (AI-assisted)	78.6% [60.5–89.8]	82.1% [64.4–92.1]	71.0% [53.4–83.9]	69.0% [50.8–82.7]	67.7% [50.1–81.4]	69.2%
NPV (unaided)	92.3% [81.8–97.0]	94.4% [84.9–98.1]	85.7% [73.3–92.9]	84.3% [72.0–91.8]	82.0% [69.2–90.2]	84.0%
NPV (AI-assisted)	92.6% [82.4–97.1]	94.4% [84.9–98.1]	92.2% [81.5–96.9]	88.7% [77.4–94.7]	90.2% [79.0–95.7]	90.4%

AI, artificial intelligence; AUROC, area under the receiver operating characteristic curve; NPV, negative predictive value; PPV, positive predictive value.

AUROC values are presented with 95% confidence intervals (CIs) in brackets. Sensitivity, specificity, PPV, and NPV are reported as percentages, with raw counts in parentheses and CIs in brackets. ∆AUROC represents the difference between unaided and AI-assisted performance. *P*-values for AUROC were calculated using DeLong’s test; *P*-values for sensitivity and specificity were calculated using McNemar’s test. R1 and R2 are senior radiologists with 15 and 12 years of experience, respectively; R3 is a junior radiologist; E1 and E2 are emergency physicians. A *P*-value < 0.05 was considered statistically significant.

Interobserver agreement improved from κ = 0.49 (moderate) in the unaided session to κ = 0.61 (substantial) with AI assistance (Fig. 5B). Diagnostic confidence scores also increased across all junior readers during the AI-assisted session (mean Δ = + 0.8; *P* < 0.01), while confidence remained unchanged among senior radiologists (Table 4).

**Table 4:** Diagnostic confidence scores with and without AI assistance.

Metric	R1	R2	R3	E1	E2	Mean (junior readers)
Experience (years)	15	12	2	5	3	3.3
Confidence scores (unaided)	4.5 ± 0.6	4.6 ± 0.5	3.6 ± 0.8	3.4 ± 0.7	3.2 ± 0.9	3.4
Confidence scores (AI-assisted)	4.6 ± 0.5	4.7 ± 0.5	4.4 ± 0.6	4.1 ± 0.6	4.1 ± 0.6	4.2
∆Confidence	0.1	0.1	0.8	0.7	0.9	0.8
*P*-value	0.682	0.700	0.006	0.009	0.005	< 0.01

Mean diagnostic confidence scores (± standard deviation) for each reader before and after AI assistance. Confidence was rated on a 5-point Likert scale (1 = very uncertain, 5 = very confident). ΔConfidence indicates the absolute change between unaided and AI-assisted conditions. *P*-values were calculated using the Wilcoxon signed-rank test. Statistically significant differences (P < 0.05) are shown in bold.

### Error analysis

**Figure 6.**
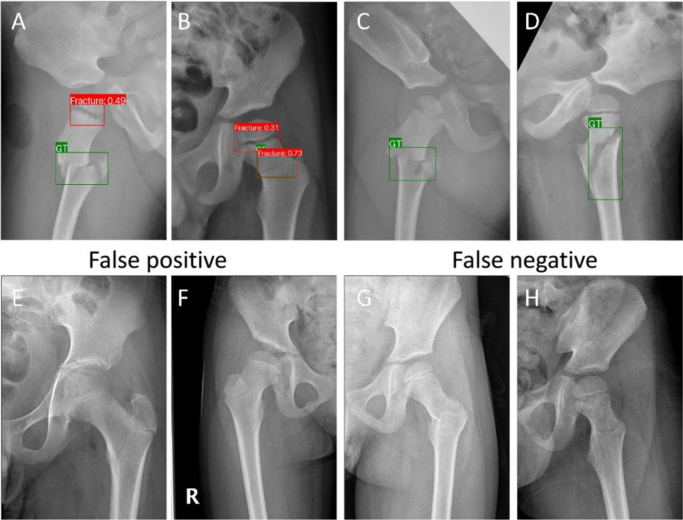
Representative AI and reader failure cases from the external test set. (A–B) AI false positives: The model incorrectly labeled normal growth plates (physes) as femoral neck fractures, generating erroneous bounding boxes (red). (C–D) AI false negatives: Subtle fracture lines were missed due to low image quality or incomplete field of view, resulting in no detection despite visible abnormalities. (E–F) Reader false positives: In unaided sessions, both a junior radiologist (E) and an emergency physician (F) misclassified normal developmental features as fractures. (G–H) Reader false negatives: Basicervical fractures located near the intertrochanteric region were overlooked, likely due to anatomical complexity and minimal displacement. Ground truth annotations are shown in green; AI-generated bounding boxes are shown in red. AI, artificial intelligence.

Post hoc qualitative analysis of the external test set revealed distinct but complementary error patterns between the AI model and human readers (Fig. 6). AI false positives (*n* = 5) frequently involved high-confidence misinterpretation of normal developmental features. In Figure 6A, the model erroneously flagged a radiolucent groove near the acetabulum. In Figure 6B, open physes and metaphyseal contours were mistaken for fracture lines, with predicted confidence scores exceeding 0.73. False negatives (*n* = 5) primarily involved subtle or nondisplaced fractures without clear cortical disruption, often obscured by overlapping anatomy or limited projection (Fig. 6C, D).

Reader errors followed similar patterns. False positives were commonly due to overinterpretation of physiologic variants, such as mild physeal irregularity or acetabular margin asymmetry (Fig. 6E and F). False negatives were most often observed in minimally displaced basicervical fractures, appearing as faint lucencies or subtle cortical irregularities, and were frequently missed in single-view examinations (Fig. 6G, H).

## Discussion

Pediatric femoral neck fractures are rare but diagnostically challenging injuries, particularly in children under 8 years of age. In this multicenter study, we developed and externally validated a YOLOv11-based deep learning model for fracture detection on pediatric hip radiographs. The model demonstrated consistently high diagnostic performance across internal and external datasets, and maintained robust sensitivity and localization accuracy despite variability in imaging projections and skeletal maturity[26].

A key clinical challenge lies in differentiating true fractures from normal developmental anatomy. In young children, the incompletely ossified proximal femur – including cartilaginous femoral heads, open physes, and apophyseal centers – can mimic fracture lines on radiographs. Our qualitative analysis revealed that AI and human readers exhibited distinct but complementary error modes: the model was more likely to overcall normal variants as fractures, while human readers were prone to missing subtle or nondisplaced fractures. These findings support the potential for AI-human collaboration to improve diagnostic reliability in complex pediatric trauma cases.

AI assistance significantly improved diagnostic accuracy, interobserver agreement, and diagnostic confidence among junior readers, but had minimal effect on senior radiologists. These findings are consistent with prior studies on pediatric fracture detection and suggest that AI may be most beneficial in settings lacking subspecialty expertise[27]. In general emergency departments or low-resource hospitals – where pediatric musculoskeletal experience may be limited – AI tools could serve as real-time decision support, reducing diagnostic variability and improving care quality for rare but high-stakes conditions[28].

While the overall model performance was strong, the clinical consequences of diagnostic errors must be considered. AI false positives may lead to unnecessary referrals or imaging, but false negatives – particularly missed nondisplaced fractures – carry greater risk due to potential delays in treatment. Future model refinements should prioritize minimizing false-negative errors, especially in cases with limited-view imaging or ambiguous skeletal features.

This study has several limitations. First, to address the rarity of pediatric femoral neck fractures, we utilized an enriched dataset with a fracture prevalence (34.3%–59.6%) significantly higher than that of a routine emergency population. While essential for model training, this enrichment introduces spectrum bias, meaning the reported PPV is likely higher than in a general, low-prevalence clinical setting. Thus, the model is intended for symptomatic, high-risk patients rather than unselected screening. Second, the external test set sample size limited the granularity of our analyses. Specifically, the relatively small number of fracture-positive cases and the scarcity of paired views (*n* = 12) precluded statistically meaningful subgroup analyses by age, fracture subtype, or view type (AP vs. Frog-leg). This limits our ability to quantify the specific value of lateral views in this workflow. Third, regarding the reference standard, although we utilized clinical follow-up and CT to confirm diagnoses, not all patients underwent advanced imaging, introducing potential verification bias. Furthermore, despite follow-up, there remains a possibility of residual error where subtle, nondisplaced fractures could theoretically heal without radiographic callus. Fourth, regarding the statistical methodology for the reader study, we utilized a simplified analysis approach (DeLong’s test) rather than a fully crossed Multi-Reader Multi-Case variance analysis framework. While effective for quantifying specific reader improvement, this approach may not fully model the random effects of reader variability, potentially limiting generalization to the broader clinician population. Finally, although the model demonstrated real-time inference speed, it was not prospectively integrated into the clinical workflow, and its effects on efficiency and clinical outcomes remain to be evaluated.

Future work should include prospective trials incorporating AI into routine clinical practice, ideally through seamless PACS integration or triage pipelines. Incorporating multi-view imaging, multimodal clinical data, and age-specific anatomical priors may further enhance model performance. In addition, explainable outputs and structured reporting may enhance clinician trust and facilitate adoption in real-world settings.

## Conclusion

In conclusion, this multicenter study demonstrates that a YOLOv11-based object detection model can accurately identify pediatric femoral neck fractures on hip radiographs and meaningfully augment clinical decision-making. When deployed as a diagnostic aid, the model improves accuracy, consistency, and confidence among less experienced readers. These findings support the adoption of real-time AI tools in pediatric trauma care, particularly for rare and high-consequence injuries in resource-limited or high-throughput environments.

## Data Availability

Deidentified data supporting the findings of this study are available from the corresponding author upon reasonable request. Data sharing will be subject to institutional review and a data use agreement.

## Research registration unique identifying number (UIN)

This trial is registered with the Chinese Clinical Trial Registry ChiCTR2300075384. (https://www.chictr.org.cn/bin/project/edit?pid=205818)
